# Genetic variation near *ROBO1* is associated with craniofacial microsomia and related phenotypes in the Finnish population

**DOI:** 10.1093/hmg/ddag020

**Published:** 2026-04-07

**Authors:** Laura Kaprio, Anu Kiukkonen, Emma Juuri, David P Rice, Hanna M Ollila, Satu Strausz

**Affiliations:** Cleft Palate and Craniofacial Centre, Department of Plastic Surgery, University of Helsinki and Helsinki University Hospital, Park Hospital, Stenbäckinkatu 11, 00029 HUS, Helsinki, Finland; Department of Oral and Maxillofacial Diseases, Head and Neck Center University of Helsinki and Helsinki University Hospital, Park Hospital, Stänbackinkatu 11, 00029 HUS, Helsinki, Finland; Cleft Palate and Craniofacial Centre, Department of Plastic Surgery, University of Helsinki and Helsinki University Hospital, Park Hospital, Stenbäckinkatu 11, 00029 HUS, Helsinki, Finland; Orthodontics, Department of Oral and Maxillofacial Disease, Faculty of Medicine, University of Helsinki and Helsinki University Hospital, Haartmaninkatu 1A, 00290, Helsinki, Finland; Orthodontics, Department of Oral and Maxillofacial Disease, Faculty of Medicine, University of Helsinki and Helsinki University Hospital, Haartmaninkatu 1A, 00290, Helsinki, Finland; Institute for Molecular Medicine Finland, Helsinki Institute of Life Science, University of Helsinki, Tukholmankatu 8, 00290, Helsinki, Finland; Broad Institute of MIT and Harvard, 415 Main Street, Cambridge, MA 02142, United States; Centre for Genomic Medicine, Massachusetts General Hospital, 185 Cambridge Street, Boston, MA 02114, United States; Anesthesia, Critical Care, and Pain Medicine, Massachusetts General Hospital and Harvard Medical School, 55 Fruit Street, Boston 02114, MA, United States; Cleft Palate and Craniofacial Centre, Department of Plastic Surgery, University of Helsinki and Helsinki University Hospital, Park Hospital, Stenbäckinkatu 11, 00029 HUS, Helsinki, Finland; Department of Oral and Maxillofacial Diseases, Head and Neck Center University of Helsinki and Helsinki University Hospital, Park Hospital, Stänbackinkatu 11, 00029 HUS, Helsinki, Finland; Institute for Molecular Medicine Finland, Helsinki Institute of Life Science, University of Helsinki, Tukholmankatu 8, 00290, Helsinki, Finland; Broad Institute of MIT and Harvard, 415 Main Street, Cambridge, MA 02142, United States; Centre for Genomic Medicine, Massachusetts General Hospital, 185 Cambridge Street, Boston, MA 02114, United States; Anesthesia, Critical Care, and Pain Medicine, Massachusetts General Hospital and Harvard Medical School, 55 Fruit Street, Boston 02114, MA, United States

**Keywords:** ROBO1, GWAS, Craniofacial microsomia, Microtia

## Abstract

Craniofacial microsomia (CFM) encompasses a phenotypic continuum of congenital anomalies ranging from isolated microtia to more complex manifestations within the oculo-auriculo-vertebral spectrum, including Goldenhar syndrome, reflecting abnormal development of first and second pharyngeal arch-derived structures. While several genetic susceptibility loci have been reported, population-based evidence in individuals of European ancestry remains limited. Using nationwide data from FinnGen in the Finnish founder population, we identified a genome-wide significant association at a conserved intergenic locus near *ROBO1*, extending previous findings to a European ancestry cohort. The lead variant, rs62256696, lies within a regulatory region active in human embryonic craniofacial tissues during early development and shows concordant association with previously reported *ROBO1* signals from non-European populations. Genetic correlation analyses demonstrated strong shared genetic architecture between CFM and auditory developmental phenotypes, consistent with the defined phenotypic continuum. Together, these findings extend previous observations to a new population context and support a role for regulatory variation at the *ROBO1* locus in early craniofacial morphogenesis and auditory system development underlying craniofacial and auditory malformations.

## Introduction

Craniofacial microsomia (CFM) is a heterogeneous spectrum of congenital anomalies arising from abnormal development of structures derived from the first and second pharyngeal arches. It includes a wide range of phenotypic presentations, from isolated microtia, congenital malformation of the external ear that ranges from minor auricular anomalies to complete absence (anotia) [1, 2] to more extensive craniofacial malformations involving the mandible, maxilla, dentition, external auditory canal, eyes, lips, tongue, palate, and vertebrae [3, 4].

Microtia frequently co-occurs with external auditory canal atresia and may present either as an isolated anomaly or as part of broader craniofacial anomalies. Within the CFM spectrum, microtia is considered a mild phenotypic manifestation, whereas more extensive anomalies often involving ocular and vertebral defects represent more severe expressions classified within the oculo-auriculo-vertebral spectrum (OAVS), with Goldenhar syndrome representing one of its more recognizable clinical presentations [2, 4–6]. These conditions converge on disrupted pharyngeal arch morphogenesis through heterogeneous mechanisms. In some cases, perturbations affecting cranial neural crest viability/survival and differentiation contribute directly (with migration defects not uniformly implicated) [7]. In contrast, in others the initiating perturbation resides in adjacent epithelial programs particularly head ectoderm (e.g. *FOXI3, GATA3, FGF3, SHROOM3*) [8–12] that secondarily alters neural crest behavior via disrupted epithelial-mesenchymal signaling. For example, spliceosomal factors such as *SF3B2* have likewise been linked to altered neural crest viability and differentiation [7]. Furthermore, recent genetic studies have expanded the CFM spectrum and implicated both coding and regulatory mechanisms. Pathogenic variants in *FOXI3* have been shown to cause one form of CFM, supporting a role for disrupted epithelial programs and epithelial-mesenchymal signaling during early craniofacial development [10]. In addition, copy-number variation affecting long-range regulatory elements near *HMX1* has been associated with isolated microtia, highlighting that non-coding structural variation can contribute to auricular malformations [13].

While CFM covers a broad range of phenotypes, microtia can also occur as a feature of well-characterized monogenic syndromes such as Treacher Collins, CHARGE, and DiGeorge syndromes. These syndromes represent distinct genetic entities with known high-penetrance mutations and are typically considered separate from the broader CFM spectrum [2, 14]. In addition, genetic correlations between CFM, malignant lymphoma, thyroid cancer, and chronic obstructive pulmonary disease have been demonstrated in Asian populations, revealing shared pleiotropic risk loci and genes [15].

The prevalence of microtia, as a recognizable and often recorded manifestation within the CFM spectrum, varies across populations, ranging from 0.83 to 17.4 per 10 000 live births globally [16–18], and 4.34 per 10 000 in Finland [18]. Estimates for the overall prevalence of CFM are somewhat lower, typically ranging between 1 and 5 per 10 000 live births [19]. Diagnostic ambiguity, particularly in milder cases, may contribute to variation in reported prevalence [4].

Despite its clinical significance, the genetic architecture of CFM remains incompletely understood. A genome-wide association study (GWAS) in Han Chinese individuals identified multiple loci associated with CFM, including regions near *ROBO1, GATA3, FGF3*, and *SHROOM3* [19]. More recently, a GWAS conducted in Latin American individuals of Amerindigenous ancestry identified a microtia-associated locus within the intergenic region between *ROBO1* and *ROBO2* [8], further supporting the role of this region in craniofacial development. However, most of these studies have focused on non-European populations, and large-scale analyses in European cohorts remain limited.

This study addresses this gap by leveraging FinnGen, a nationwide biobank-based initiative that integrates genome-wide genotyping data with comprehensive longitudinal health records from the Finnish population. The unique genetic profile of this founder population characterized by reduced allelic diversity and extended linkage disequilibrium provides a powerful framework for gene discovery and signal refinement, especially for rare phenotypes such as CFM. By combining this resource with phenome-wide and genome-wide correlation analyses, our study aims to expand the current understanding of the genetic and developmental basis of CFM and its phenotypic continuum [20–22].

## Results

### GWAS identifies a genome-wide significant locus near *ROBO1*

**Figure 1:**
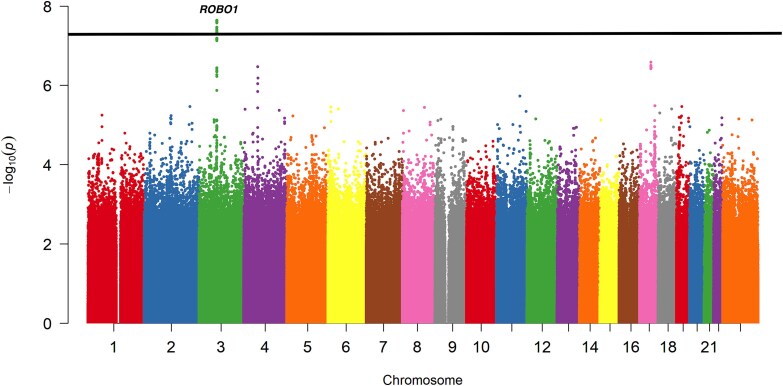
Manhattan plot of a genome-wide association study for craniofacial microsomia (CFM) in individuals of Finnish ancestry (N cases = 82, N controls = 500 104). A genome-wide significant locus was detected on chromosome 3 (lead SNP: rs62256696, *P* = 2.27 × 10^−8^) near the *ROBO1* gene. The y-axis represents –log₁₀(p) values of association, and the genome-wide significance threshold (*P* = 5 × 10^−8^) is marked by the horizontal black line.

We conducted a GWAS of CFM including 82 cases and 500 104 controls utilizing data from the FinnGen cohort (Fig. 1). Among the cases, 52 (63.4%) were female and 30 (36.6%) were male, while the control group included 281 857 (56.4%) females and 218 247 (43.6%) males. The sex distribution did not differ significantly between cases and controls (χ^2^ = 1.4, *P* = 0.24). The analysis identified one genome-wide significant locus on chromosome 3, (rs62256696, beta = 0.96, se = 0.17, *P* = 2.27 × 10^−8^). Fine-mapping defined a 95% credible set comprising 45 variants near the *ROBO1* region, with the lead variant showing a posterior probability of 2.95% (Supplementary Table 1). The lead variant was in significant LD with rs13089920 (r^2^ = 0.33, *P* < 0.0001) and rs1444472 (r^2^ = 0.58, *P* < 0.0001), previously reported in association with CFM [19] and microtia [8], respectively.

Given the limited number of cases, we additionally report loci reaching suggestive significance (*P* < 1 × 10^−6^). Two such loci were observed on chromosomes 4 and 17. The lead variants were rs140478588 (beta = 2.04, se = 0.40, *P* = 3.39 × 10^−7^), annotated as an intronic variant in *ADGRL3*, and rs1280525466 (β = 4.92, se = 0.96, *P* = 2.59 × 10^−7^), annotated as a downstream variant of *SPAG9*. Both signals involve low-frequency alleles, particularly rs1280525466, which was rare in controls (alt = 0.000138) but enriched among cases (alt = 0.0183) and the corresponding effect estimates may be sensitive to small numbers of carriers. Full variant-level results for both regions are provided in Supplementary Table 2.

**Figure 2:**
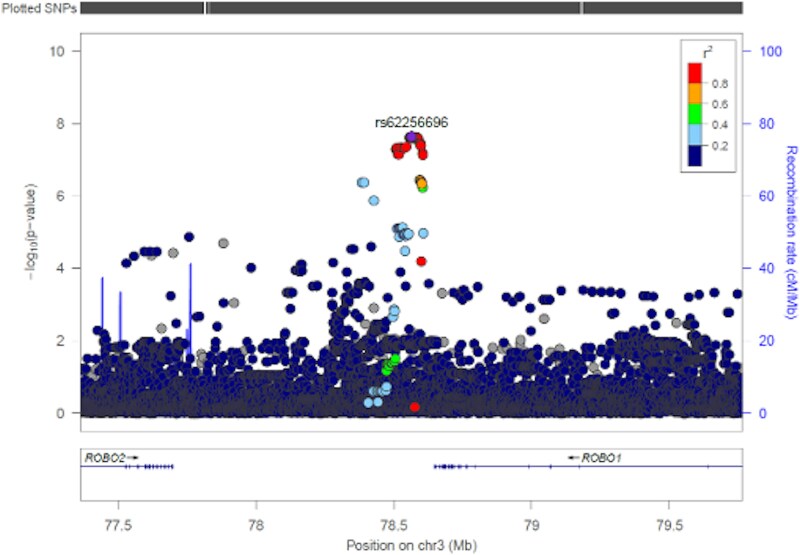
Regional association plot for the lead locus associated with craniofacial microsomia (CFM), centered on rs62256696 at the *ROBO1/ROBO*2 interval. The lead SNP reaches genome-wide significance (*P* = 2.27 × 10^−8^). SNPs are colored by linkage disequilibrium (LD) with the lead SNP. The plot highlights a high-LD block in the intergenic region between *ROBO1* and *ROBO2* locus.

A regional association plot of the chromosome 3 locus (Fig. 2) demonstrated that the lead SNP, rs62256696, is located within a high-LD block in the intergenic interval between *ROBO1* and *ROBO2*, with *ROBO1* being the nearest protein-coding gene, and several surrounding variants (r^2^ > 0.8) showing similarly strong association signals.

**Figure 3:**
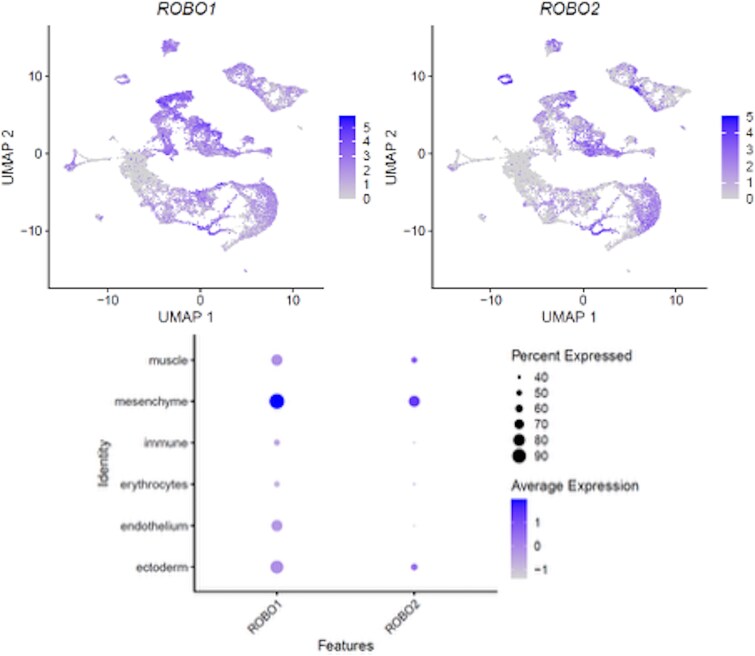
*ROBO1* and *ROBO2* are expressed during early human craniofacial development. UMAP feature plots (top) show log-normalized *ROBO1* (left) and *ROBO2* (right) expression in publicly available fetal human craniofacial (developing face) single-nucleus RNA-seq data (4–8 post-conception weeks) [23]. The dot plot (bottom) summarizes *ROBO1/ROBO2* expression across major annotated craniofacial cell populations; dot size indicates the fraction of expressing cells and color indicates average expression.

Publicly available single-nucleus RNA-seq data [23] from fetal human craniofacial tissues showed *ROBO1* and *ROBO2* expression across multiple developmental cell populations, including mesenchymal cells (Fig. 3). These findings support developmental relevance of the *ROBO1/ROBO2*-associated region for craniofacial morphogenesis.

**Figure 4:**
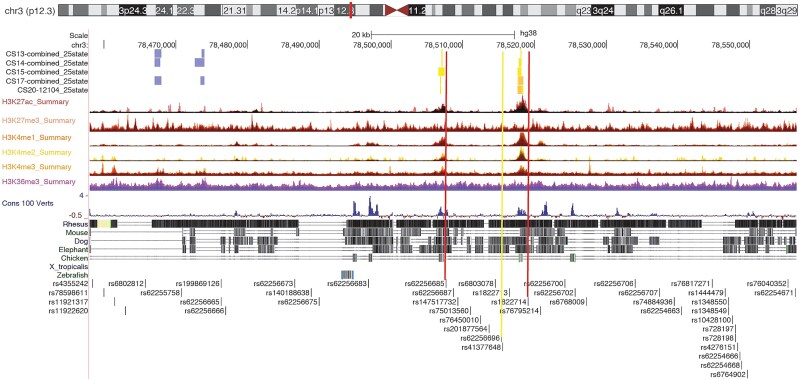
UCSC genome browser view of the human genome GRCh38/hg38, associated region downstream of *ROBO1*with rs62256696 and other credible variants. Tracks 1–5 show enhancer marks in Carnegie stage 13–20 and tracks 6–18 show combined signal marks of human craniofacial tissue [22]. Tracks 12 shows the multiple alignments of 100 vertebrate species and tracks 13–19 shows evolutionary conservation. The last track shows the credible variants. Yellow line indicates the top variant. Red lines indicate rs62256685 and rs1822714 that overlap multispecies conserved sequences.

To evaluate the epigenetic profile of the variants in the credible set further, we analyzed their positions relative to chromatin marks indicative of open chromatin and active enhancer function (Fig. 4). The top variant, rs62256696, along with variants in high linkage disequilibrium (LD), was located in the intergenic region between *ROBO1* and *ROBO2*. Two variants in LD with rs62256696, rs62256685 (r^2^ = 0.99) and rs1822714 (r^2^ = 1), resided within two distinct regions exhibiting enhancer activity throughout early developmental stages of human craniofacial tissue. Furthermore, both variants overlapped multispecies conserved sequences and were assigned as likely conserved by GerpS score (rs1822714, Supplementary Table 3), or by five conservation scores within primates and mammals (rs62256685; mamPhyloP, GerpN, GerpS, priPhyloP, verPhyloP) (Supplementary Table 4).

### Clinical CodeWAS enrichment patterns in craniofacial microsomia cases and *ROBO1* risk variant homozygotes

In our data, rs62256696 Alt/Alt homozygosity was present in 6 of 82 CFM cases (7.32%) and in 5304 of 500 104 non-CFM individuals (1.06%). To characterize the broader clinical profile associated with homozygosity, we performed a CodeWAS comparing rs62256696 Alt/Alt homozygotes (N = 5310) with 10× age- and sex-matched controls. In parallel, a case–control CodeWAS compared CFM cases (N = 82) with 10× age- and sex-matched controls. Table 1 summarizes clinical codes significantly enriched in both analyses (*P* < 0.05). The strongest concordant enrichments were observed in auditory system-related evaluations and rehabilitation, including ear microscopy, audiometry, and hearing-aid related procedures, with additional overlap in speech therapy, genetic counselling, and congenital malformation-related codes. These findings indicate that similar clinical features and treatment codes are observed in a broader group of individuals carrying the *ROBO1* risk genotype than among those captured by a clinical CFM diagnosis alone (Table 1).

**Table 1:** Clinical CodeWAS associations enriched both in individuals diagnosed with craniofacial microsomia (CFM) and in homozygous (alt/alt) carriers of *ROBO1* risk variants.

**Domain**	**Phenotype**	**OR (CFM)**	**P (CFM)**	**OR (Alt/Alt)**	**P (Alt/Alt)**
Audiology/Hearing	Audiometric test	5.8	1.42 × 10^−9^	22.94	6.41 × 10^−240^
	Examination of ears and hearing	9.77	1.85 × 10^−17^	7.25	5.65 × 10^−163^
	Ear microscopy	9.41	3.84 × 10^−11^	6.64	3.33 × 10^−89^
	Fitting and adjustment of hearing aid	14.84	2.99 × 10^−5^	9.29	2.32 × 10^−66^
	Presence of external hearing-aid	10.73	2.43 × 10^−4^	7.98	1.78 × 10^−50^
	Adjustment or review of hearing aid	41.27	8.24 × 10^−14^	6.54	4.49 × 10^−44^
Craniofacial and oropharyngeal procedures	Other minor procedures in surgery of teeth, jaws, mouth and pharynx	2.65	2.71 × 10^−2^	13.31	4.15 × 10^−62^
ENT procedures	Rhinopharyngoscopy	3.1	1.14 × 10^−2^	5.93	1.51 × 10^−60^
	Laryngoscopy	4.27	3.69 × 10^−3^	5.09	9.63 × 10^−42^
	Other minor procedure in surgery of ear, nose and larynx	5.81	7.49 × 10^−4^	5.66	3.08 × 10^−27^
	Insertion of ventilating tube through tympanic membrane	4.75	1.51 × 10^−5^	4.43	2.02 × 10^−15^
Speech	Speech therapy	9.24	1.40 × 10^−4^	4.69	8.10 × 10^−19^
	Speech and linguistic disorders	3.06	8.67 × 10^−3^	7.04	7.22 × 10^−17^
Genetic counselling	Genetic counselling	5.17	4.42 × 10^−6^	15.59	4.79 × 10^−92^
Cardiac imaging	Extensive structural and functional ultrasound examination of heart	2.17	1.63 × 10^−2^	13.21	<1 × 10^−300^
	Standard cardiac ultrasound examination	2.73	4.89 × 10^−3^	11.65	2.00 × 10^−251^
Congenital malformations	Congenital malformations of heart and great arteries	10.87	5.48 × 10^−10^	5.91	1.13 × 10^−19^

Odds ratios (OR) and p-values are shown for clinical codes significantly enriched among both individuals diagnosed with craniofacial microsomia and homozygous carriers (Alt/Alt) of the associated *ROBO1* variants, as identified using a CodeWAS approach.

We next performed linkage disequilibrium score regression (LDSC) to assess genetic correlations between CFM and other traits in FinnGen. Strong genetic correlations were observed with auditory system-related phenotypes including congenital malformations of the ear causing hearing impairment (rg = 0.44, *P* = 8.1 × 10^−6^) and congenital absence or narrowing of the external auditory canal (rg = 0.37, *P* = 7.2 × 10^−5^). These findings are consistent with the known clinical and developmental involvement of auditory structures within the CFM spectrum.

### Consistency with prior reports

Among the previously reported genome-wide significant loci, SNPs located at the *ROBO1* locus rs13089920 (*P* =  1.11 × 10^−5^), and rs1444472 (*P* = 4.38 × 10^−7^) showed replication in our Finnish dataset with consistent effect directions. In contrast, we did not observe nominally significant associations at the other previously reported loci in our cohort. However, given the substantially smaller number of cases in FinnGen, these comparisons are underpowered and should not be interpreted as definitive non-replication (Table 2).

**Table 2:** Genome-wide significant loci associated with craniofacial microsomia (CFM) and microtia across different populations and studies.

**Population/Study**	**Lead SNP**	**Gene/Region**	**OR**	**P-value**	**FinnGen OR**	**FinnGen P-value**
ROBO1 locus SNPs						
Han Chinese CFM GWAS	rs13089920	ROBO1 locus	5.2	2.15 × 10^−120^	1.89	1.11 × 10^−5^
Latin American microtia GWAS	rs1444472	ROBO1 locus	4.5	2.35 × 10^−8^	2.2	4.38 × 10^−7^
Other genome-wide significant loci						
Han Chinese CFM GWAS	rs17802111	EPAS1	1.48	9.57 × 10^−18^	0.88	4.01 × 10^−1^
Han Chinese CFM GWAS	rs7420812	PARD3B, NRP2	1.33	6.74 × 10^−10^	1.22	3.34 × 10^−1^
Han Chinese CFM GWAS	rs3754648	GBX2	1.39	5.09 × 10^−13^	1.2	2.68 × 10^−1^
Han Chinese CFM GWAS	rs10905359	FGF3	0.76	5.11 × 10^−9^	0.84	2.99 × 10^−1^
Han Chinese CFM GWAS	rs11263613	ARID3B	1.68	3.61 × 10^−17^	0.57	3.38 × 10^−1^
Han Chinese CFM GWAS	rs10459648	KLF12	0.63	1.05 × 10^−23^	1.12	6.37 × 10^−1^
Han Chinese CFM GWAS (left-sided)	rs17090300	SHROOM3	2.31	1.04 × 10^−11^	1.16	6.90 × 10^−1^

OR = odds ratio.

## Discussion

We performed a genome-wide association study of CFM in a large, well-characterized European founder population and identified a significant locus at the *ROBO1/ROBO2* intergenic interval. The association signal overlaps regulatory annotations at this locus, and clinical correlations in variant carriers suggest broader developmental relevance beyond diagnosed CFM. Together, our findings extend previous observations to a new population context and prioritize the *ROBO1* locus for follow-up, although larger studies will be required to delineate the broader genetic architecture of CFM.

### Genome-wide association signals at the *ROBO1* locus

Our genome-wide association analysis identified a statistically significant locus on chromosome 3 near the *ROBO1* gene region. The lead variant, rs62256696, reached genome-wide significance and was located within a high LD block overlapping *ROBO1*. Fine-mapping yielded a 95% credible set of 45 variants, with the lead SNP showing the highest posterior probability. Notably, this region also includes rs1444472, a variant previously implicated in microtia in an Amerindigenous population through a study of a tandem repeat between *ROBO1* and *ROBO2* [8]. Quiat et al. also provided evidence that this intergenic interval harbors an expanded complex tandem repeat, which is a strong candidate causal susceptibility variant class [8]. Because complex tandem repeats are not reliably captured by array-based genotyping and imputation, we could not directly characterize repeat structure in FinnGen; thus, our association likely tags a broader regulatory haplotype within a shared *ROBO1/ROBO2* susceptibility interval. Similarly, a prior GWAS conducted in Han Chinese individuals identified *ROBO1* as one of several loci associated with CFM [19].

These earlier studies support the involvement of the *ROBO1* region in craniofacial development across multiple populations. Our findings now extend this evidence to individuals of European ancestry, providing population-level replication and emphasizing the broader relevance of this regulatory locus. The associated region lacks coding variants but overlaps predicted regulatory elements, suggesting that the signal may act through modulation of *ROBO1* expression during early craniofacial development. Recent work has also implicated additional genes in the CFM spectrum, including *SF3B2* [7], *CHAF1A* [24], *SHROOM3* [9], and *FOXI3* [10], particularly in familial and rare-variant contexts, underscoring genetic heterogeneity and likely multiple developmental mechanisms.

### Developmental context of the *ROBO1/ROBO2* locus

To place the *ROBO1/ROBO2* association in a mechanistically relevant context, we examined publicly available single-nucleus RNA-seq data from fetal human craniofacial tissues (developing face, 4–8 post-conception weeks) [23]. *ROBO1* and *ROBO2* show expression during early craniofacial development across multiple cell populations, including mesenchymal cells. As craniofacial mesenchyme at these stages is largely derived from cranial neural crest, these fetal expression data provide developmental context for the *ROBO1/ROBO2* locus in craniofacial morphogenesis [25].

*ROBO1* is a receptor in the Slit–Robo signaling pathway, which has established roles in guidance signaling and tissue patterning during embryogenesis and has been implicated in craniofacial developmental processes [25]. Altered *ROBO1* expression or function could therefore disrupt neural crest cell patterning and lead to structural anomalies such as mandibular hypoplasia, ear malformations, and facial asymmetry characteristic of CFM. Moreover, studies in animal models have demonstrated that Slit-Robo signaling is critical for branchial arch development, the embryological origin of the mandible and external ear [26, 27].

Consistent with this interpretation the lead variant rs62256696 and its linked variants reside in an intergenic region between *ROBO1* and *ROBO2* that is marked by enhancer activity in human craniofacial tissues during a critical early developmental window (Carnegie stages 13–20). These regions overlap multispecies conserved sequences, indicating evolutionary constraint and supporting their potential functional relevance in craniofacial development.

### Concordant clinical patterns in CFM and *ROBO1* risk variant carriers support a shared developmental pathway

Beyond the genetic association, we observed phenotypic overlap between individuals with clinically diagnosed CFM and homozygous carriers of the associated *ROBO1* variant, supporting a shared developmental origin. Traits such as ear microscopy, audiometry, and congenital malformations were enriched in both groups, indicating that the locus may contribute not only to overt CFM but also to a broader range of craniofacial and auditory features. In addition, enrichment of speech therapy and genetic counselling codes supports involvement of a broader craniofacial-auditory developmental care pathway rather than isolated otologic findings*.* The presence of CFM-adjacent phenotypes in individuals without a formal diagnosis suggests that the associated variant may act through regulatory mechanisms influencing gene expression during craniofacial development. This pattern is consistent with variable expressivity and with incomplete capture of granular craniofacial features in registry-based data, where milder or partial presentations may not meet formal diagnostic thresholds. Consistent with these clinical overlaps, genetic correlation analyses using LDSC further supported shared heritable architecture between CFM and congenital ear malformations causing hearing impairment.

Together, these findings support a model in which genetic variation at the *ROBO1* locus alters neural crest development, contributing to a spectrum of craniofacial phenotypes ranging from isolated microtia to broader CFM features. While this locus has been associated with craniofacial anomalies in non-European populations, our findings demonstrate its relevance in a European cohort, thus extending previous observations to a new ancestral context.

### Limitations

Several limitations should be noted. Case ascertainment was registry-based and relied on diagnostic codes for microtia and/or Goldenhar syndrome, which limits harmonized feature-level phenotyping (e.g. laterality, severity grading, and individual clinical signs) and restricts detailed genotype–phenotype stratification. The number of cases (N = 82) limits GWAS power to detect modest effects and additional loci; therefore, suggestive associations often involving low-frequency alleles should be considered hypothesis-generating and require independent replication. In addition, complex tandem repeats such as the *ROBO1/ROBO2* repeat highlighted by Quiat et al. are not reliably captured by array genotyping and imputation and therefore could not be directly assessed in our dataset [8]. Functional interpretation is based on publicly available regulatory annotations and fetal craniofacial expression resources without new experimental validation. Although the *ROBO1/ROBO2* region has been implicated across multiple populations, differences in allele frequencies and linkage disequilibrium across cohorts may influence fine-mapping resolution and the detection of additional signals, motivating follow-up in larger and diverse datasets.

## Materials and methods

### Cohort description

FinnGen is a large-scale research project that has combined comprehensive genomic and health data from 500 000 Finnish individuals. Launched in collaboration with several Finnish biobanks and research institutions, FinnGen endeavors to identify genetic variants associated with common diseases, enabling the development of more targeted and effective treatments. The project harnesses the power of high-throughput genomic sequencing technologies to analyze the genomes of tens of thousands of participants, contributing to the elucidation of complex genetic mechanisms underlying diseases such as cardiovascular disorders, diabetes, and cancer [20].

### Phenotype definition

**Figure 5:**
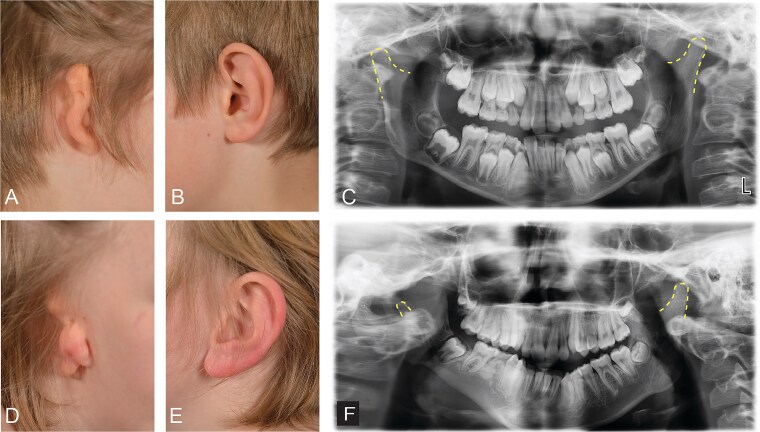
Clinical spectrum of craniofacial microsomia, ranging from isolated microtia to Goldenhar syndrome. (A–C) unilateral microtia in an 8-year-old male showing malformation of the pinna (A), the unaffected contralateral ear (B), and a panoramic radiograph obtained at age 10 (C). (D–F) Goldenhar syndrome in a 13-year-old male showing a malformed pinna with absence of the external auditory canal (D), the unaffected contralateral ear (E), and a radiograph revealing severe condylar hypoplasia on the affected side compared with the contralateral condyle, resulting in marked mandibular asymmetry (F). Yellow dashed line represents the outline of the condylar process.

The diagnosis of CFM was based on ICD codes (ICD-10: Q17.2; ICD-9: 7442B for microtia and ICD-10: Q87.03 for Goldenhar), which were obtained from the Finnish National Hospital Discharge and Primary Care Registries, Causes of Death Registry and Malformation Registry. Among the 82 cases, 67 had microtia codes only, 8 had Goldenhar codes only, and 7 had both. Representative clinical presentations of the CFM spectrum, ranging from microtia to Goldenhar syndrome, are shown in Fig. 5.

### Genotyping and imputation

Genotyping in the FinnGen cohort was performed by using Illumina (Illumina Inc., San Diego, CA, USA) and Affymetrix arrays (Thermo Fisher Scientific, Santa Clara, CA, USA) and lifted over to Genome Reference Consortium Human Build version 38 (GRCh38/hg38). Individuals with high genotype absence (>5%), inexplicit sex or excess heterozygosity (+ − 4 standard deviations) were excluded from the data. Additionally, variants that had high absence (>2%), low minor allele count (<3) or low Hardy–Weinberg Equilibrium (HWE) (P < 1 × 10–6) were removed. All individuals in the cohort were Finns and matched against the SiSu v4 reference panel (http://www.sisuproject.fi/).

Before imputation, array-genotyped samples were pre-phased with Eagle 2.3.5 using the default parameters, except the number of conditioning haplotypes, which was set to 20 000.

Genotype imputation was carried out by using the population-specific SISu v4.2 imputation reference panel with Beagle 4.1 (version 27Jan18.7e1). Post-imputation quality control involved checking the expected conformity of the imputation INFO-value distribution, MAF differences between the target dataset and the imputation reference panel and checking chromosomal continuity of the imputed genotype calls.

### Genetic analyses

Genome-wide association testing was conducted using the Regenie v2.2.4 software and the FinnGen Regenie pipeline (https://github.com/FINNGEN/regenie-pipelines). The analysis was adjusted for current age or age at death, sex, genotyping chip, genetic relationship, and the first 10 principal components. To explore the causality of genomic variations in the *ROBO1* locus related to CFM, a fine-mapping approach was employed using the SuSiE ‘Sum of Single Effects’ model [21].

To investigate the regulatory landscape of rs62256696 and variants in high linkage disequilibrium (LD), we utilized the UCSC Genome Browser (https://genome.ucsc.edu/) alongside datasets from the Gene Expression Omnibus (GEO, https://www.ncbi.nlm.nih.gov/geo/). The GEO datasets included chromatin state tracks and enhancer marks derived from human craniofacial tissues at Carnegie Stages 13–20 (CS13–20), enabling identification of candidate enhancer elements active during early developmental stages [22]. To assess evolutionary conservation, we employed the UCSC 100 Vertebrates and MultiZ alignments.

We employed Linkage Disequilibrium Score Regression (LDSC) to investigate the polygenic architecture of CFM. We estimated the contribution of common genetic variants to CFM by calculating genetic correlations between our GWAS summary statistics and over 2400 phenotypes available in the FinnGen dataset. LDSC leverages patterns of linkage disequilibrium across the genome to assess genome-wide genetic similarity between traits.

### Association analysis across health registry codes

We conducted a code-wide association study (CodeWAS) to evaluate the phenotypic spectrum associated with CFM and homozygosity for the lead *ROBO1* risk variant rs62256696 (Alt/Alt). The analysis was performed within the FinnGen dataset by systematically testing associations across ICD-coded diagnoses, procedure codes, medication purchases (ATC codes), and causes of death. For each analysis, cases (CFM cases or rs62256696 Alt/Alt homozygotes) were matched to up to 10 population controls based on age and sex. The association analyses were carried out using logistic regression models adjusted for sex and age.

## Supplementary Material

ddag020_Supplemental_Files

## Data Availability

Summary statistics of the GWAS are available at GWAS Catalog (Accession number: GCST90832113).
